# Primary Cutaneous Lymphomas and Lymphoproliferative Disorders: Current Insights for Classification and Management

**DOI:** 10.3390/cancers18183038

**Published:** 2026-09-19

**Authors:** Uğur Hatipoğlu, Mert Seyhan, Hakan Eminoglu, Turgay Ulaş, Mehmet Sinan Dal

**Affiliations:** Department of Hematology and Bone Marrow Transplantation Center, University of Health Sciences, Ankara Oncology Training and Research Hospital, Ankara 06200, Türkiye; drseyhanmert@gmail.com (M.S.); hakaneminoglu13@gmail.com (H.E.); turgayulas@yahoo.com (T.U.); dr.sinandal@gmail.com (M.S.D.)

**Keywords:** primary cutaneous lymphoma, cutaneous lymphoproliferative disorders, WHO 5th classification, International Consensus Classification (ICC), cutaneous T-cell lymphoma, cutaneous B-cell lymphoma, precision oncology, targeted therapy, radiotherapy

## Abstract

Background: Primary cutaneous lymphomas (PCLs) represent a heterogeneous group of extranodal non-Hodgkin lymphomas with distinct clinical, histological, and molecular profiles. Recent advancements in genomic profiling have led to significant revisions in diagnostic frameworks and therapeutic paradigms. Objective: This review aims to summarize the current landscape of primary cutaneous B-cell and T-cell lymphomas and lymphoproliferative disorders (LPDs), focusing on the diagnostic updates in the WHO 5th edition and the International Consensus Classification (ICC 2022), alongside evolving risk-adapted treatment strategies. Methods: A comprehensive review of the recent literature and major international consensus guidelines was conducted, evaluating diagnostic shifts, disease reclassifications, and novel therapeutic interventions across various PCL subtypes. Results: The latest WHO 5th and ICC 2022 classifications emphasize the clinical behavior of indolent entities, shifting terminology from “lymphoma” to “lymphoproliferative disorder” for conditions such as primary cutaneous acral CD8+ T-cell LPD and primary cutaneous CD4+ small/medium T-cell LPD to prevent overtreatment. Several previously provisional categories (e.g., primary cutaneous gamma/delta T-cell lymphoma and EBV-positive mucocutaneous ulcer) have achieved definitive diagnostic status. Parallel to these revisions, therapeutic management has shifted toward toxicity-reducing, risk-adapted strategies. For indolent subtypes, ultra-low-dose radiotherapy (4 Gy) and skin-directed therapies remain highly effective. In aggressive or refractory subtypes, such as Mycosis Fungoides and primary cutaneous diffuse large B-cell lymphoma, leg type (PCDLBCL, LT), precision oncology—utilizing targeted agents (e.g., brentuximab vedotin, mogamulizumab, lenalidomide, ibrutinib) and cellular/immunotherapies—is rapidly changing outcomes. Conclusions: Integrating the updated diagnostic classifications with modern risk-stratified and targeted treatment paradigms is crucial for optimizing clinical management and avoiding overtreatment in indolent PCLs while improving survival in aggressive subtypes.

## Simple Summary

Primary cutaneous lymphomas and lymphoproliferative disorders represent a biologically distinct spectrum of extranodal neoplasms that warrant different clinical approaches. Recent international consensus updates (WHO 5th edition and ICC 2022) have redefined diagnostic boundaries by downgrading several indolent entities as lymphoproliferative disorders rather than overt lymphomas to avoid unnecessary treatment. In parallel, therapeutic strategies have evolved toward toxicity-reducing, risk-adapted modalities—such as ultra-low-dose radiotherapy and skin-directed therapies for indolent disease. Lastly, targeted biologics and immunotherapies have been used to treat aggressive subtypes.

## 1. Introduction

Primary cutaneous lymphomas (PCLs) are relatively rare and heterogeneous neoplasms, derived from B and T-lymphocytes [1]. They represent the second most common extranodal non-Hodgkin lymphoma [2]. Primary cutaneous lymphomas (PCLs) are rare hematologic neoplasms with an annual Western incidence of 1 per 100,000, presenting as skin-limited disease without baseline extracutaneous spread. This cutaneous manifestation uniquely facilitates the integration of macroscopic clinical findings with histological, immunophenotypic, and genomic data—a diagnostic advantage absent in other hematologic malignancies. While classic epidemiology attributes 75–80% of PCL cases to T-cell lineages (CTCL) and 20–25% to B-cell lineages, a recent global meta-analysis has highlighted a higher CTCL frequency of 83%, dominated primarily by Mycosis Fungoides (MF) [2]. In recent years, PCL classification has changed significantly as with nodal lymphomas, and genetic studies are being used more frequently [2]. The WHO-EORTC classification of PCL was published in 2005 and revised in 2018 [3]. In 2022, the WHO 5th classification of hematolymphid tumors and International Consensus Classification (ICC) further provided new classifications, including for cutaneous lymphomas [4,5]. Both systems reflect a shared movement toward recognizing the indolent nature of certain conditions; notably, CD8+ acral T-cell lymphoma has been downgraded to better align with its indolent clinical behavior. Primary cutaneous CD4+ small/medium T-cell lymphoproliferative disorder is retained as an indolent lymphoproliferative disorder characterized by a very favorable prognosis and common presentation as a solitary nodule primarily in the head and neck area; and EBV-positive mucocutaneous ulcer (EBVMCU) is established as a distinct, circumscribed, EBV-driven entity occurring in the setting of age-related immunosenescence or immunosuppressive treatment that follows a favorable, self-limiting course often responding to the reduction in immunosuppressive therapy despite its atypical morphological features [4,5]. In the latest edition of the WHO 5th Skin Tumor classification, mature plasmacytoid dendritic cell proliferation associated with myeloid neoplasm, *ALK*-positive histiocytosis, and IEI-associated T-cell lymphoproliferative disorders have been added [6].

Similar to classification systems, treatment paradigms are constantly evolving [7]. Due to several downgrades in classification schemes, therapies need to be reshaped in parallel with changes in entity definitions and should avoid unnecessarily intensive approaches [1]. Other key updates include the omission of extensive systemic staging in favor of thorough clinical examinations for typical cases of primary cutaneous CD4+ small/medium T-cell lymphoproliferative disorder and EBV-positive mucocutaneous ulcer; furthermore, low-dose radiotherapy (4–8 Gy) represents a suitable alternative to higher doses (≥20 Gy) for PCSM-TCLPD and acral CD8+ TCLPD, whereas a 4 Gy regimen can be used for PCMZL/LPD [1]. Recent therapeutic trends in Mycosis Fungoides (MF) are characterized by the integration of newly licensed agents such as brentuximab vedotin and mogamulizumab. Furthermore, newer radiation techniques like low-dose total skin electron beam therapy (TSEB) can be useful for reducing toxicity in aggressive disease [8]. The therapeutic arsenal for aggressive subtypes like primary cutaneous diffuse large B-cell lymphoma, leg type (PCDLBCL, LT), is rapidly shifting toward precision medicine, with the integration of targeted agents such as lenalidomide, ibrutinib, and PD-1 inhibitors that exploit specific genetic vulnerabilities like *MYD88* mutations, though this testing is not standard in everyday practice. Rituximab combined with a chemo backbone remains standard [9].

In this review, we aim to review the latest literature regarding current classification and management options for cutaneous lymphomas/lymphoproliferative disorder and look into the future insights.

## 2. B-Cell Cutaneous Lymphomas (BCCL)/Lymphoproliferative Disorders (B-LPD)

### 2.1. Current Classification of BCCL and B-LPD

Primary cutaneous marginal zone lymphoma (PCMZL), primary cutaneous follicular center lymphoma (PCFCL) and primary cutaneous diffuse B-cell lymphoma, leg type (PCDLBCL-LT), are the major three types of BCCL in the WHO 5th edition of tumor classification and ICC 2022 [10]. Intravascular B-cell lymphoma and EBV-positive mucocutaneous ulcer are also recognized by both WHO and ICC, but these are less frequent neoplasms relative to marginal, follicular and large B-cell lymphomas of the skin [10]. The WHO 5th edition includes unique disorders that are missing in ICC [10]. The 5th edition of the WHO classification has replaced the historical term “cutaneous pseudolymphoma” with “cutaneous reactive T- and B-cell rich lymphoid proliferations” (rT-LP and rB-LP) to align with modern oncological nomenclature [4]. Despite their benign nature, these disorders pose a significant diagnostic challenge as they frequently harbor atypical cells that histologically and immunologically mimic malignant cutaneous T-cell (by expressing CD30) or B-cell lymphomas. Consequently, recognizing these entities is critical for preventing overtreatment. These proliferations lack IgH monoclonality or light-chain restriction, and tingible body macrophages can be seen histologically [10]. Detection of Borrelia spp. in specimens may raise suspicion of reactive lymphoid proliferation [10]. While clonality studies are complementary, accurate distinction should rely on integrated clinical, histological, immunophenotypic, and molecular assessment (Table 1).

PCFCL, the most common B-cell-derived cutaneous lymphoma, accounts for %30–60 of all BCCL [9,11,12]. The classification of primary cutaneous lymphomas are evolving towards a multidisciplinary synthesis of clinical, histological, and molecular features. PCFCL typically presents in middle-aged adults as solitary or grouped erythematous to violaceous papules and nodules localized in the head, neck, or trunk. Histologically, it consists of centrocytes and centroblasts arranged in follicular or diffuse patterns that express germinal center markers like BCL6 and CD10. Crucially, PCFCL is genetically distinct from its systemic follicular counterparts; it usually lacks *BCL2* expression and the t(14;18) translocation, instead harboring *TNFRSF14* mutations while rarely showing the *CREBBP* or *KMT2D* mutations seen in nodal disease. This marked biological diversity has led to PCFCL’s unique classification in the WHO 5th edition [4,13,14] [Table 1].

PCDLBCL, LT is an aggressive malignancy that predominantly affects elderly women and presents as rapidly progressive, often ulcerated, red-brown to blue tumors on one or both lower legs. Unlike indolent B-cell lymphomas, PCDLBCL, LT cells highly express *BCL2*, *MUM-1/IRF-4*, *FOXP1*, and IgM, suggesting the activated B-cell (ABC) subtype of systemic DLBCL. The genetic landscape is further defined by a high prevalence of *MYD88 L265P* mutations (approximately 75%) and *CD79B* mutations, which activate the NF-κB pathway similar to DLBCLs of immune-privileged sites and contribute to a poor prognosis.

In the latest 5th edition of the WHO classification and the International Consensus Classification (ICC), PCDLBCL, LT remains strictly classified as an overt lymphoma because of its inherently aggressive clinical course, high rate of extracutaneous dissemination, and significantly inferior survival rates [4,12,15].

PCMZL is an highly indolent disease with excellent prognosis; 5-year survival rates exceed 99% [2]. In the previous version of the WHO classification, PCMLZs were grouped under mucosa-associated lymphoid tissue (MALT) lymphomas. Recent studies have challenged this concept, since PCMZL is not generally associated with translocations like MALT lymphoma. Furthermore, the true malignant characteristics of PCMZL are often questioned, and there is a growing consensus toward defining PCMZL as a lymphoproliferation rather than a true lymphoma [16,17,18]. As synthesized by Zhang et al. [17], this shift in terminology was motivated by molecular and clinical evidence demonstrating that cutaneous lesions lack canonical systemic MALT lymphoma translocations (such as $t\left(11;18\right)$) and exhibit unique genetic lesions (e.g., *FAS* mutations) paired with near-perfect disease-specific survival.

The International Consensus Classification of 2022 downgraded the disease to “primary cutaneous marginal zone lymphoproliferative disorder” rather than “lymphoma” due to its low risk of systemic dissemination and remarkable favorable prognosis. The WHO 5th classification system defined PCMZL under the marginal zone lymphomas section and removed it from the MALT umbrella [4,5].

### 2.2. Current Therapeutic Approaches for BCCL and B-LPD

The management of primary cutaneous follicle center lymphoma (PCFCL) and other indolent B-cell lymphoproliferative disorders is highly risk-adapted, and appropriate staging is essential [19] [Table 2 and Table 3].

Therapeutic decisions for primary BCCLs are primarily driven by lesion characteristics, specifically lesion number, size, anatomic distribution, and TNM staging category (solitary/regional T1–T2 versus generalized cutaneous T3 disease). In addition, the presence of symptoms, documented extracutaneous (nodal or visceral) involvement, and general patient fitness critically dictate the treatment intensity, distinguishing candidates for localized approaches (such as involved-site radiation therapy, surgical excision, or active observation in asymptomatic indolent disease) from those requiring systemic chemoimmunotherapy [19].

For patients presenting with solitary lesions, low-dose radiotherapy (LDRT) at 4 Gy can be considered with excellent safety and efficacy. Local radiotherapy is highly effective, offering a 100% objective response rate, but may not be durable, with a five-year disease-free survival of 51%. Alternatively, surgical excision remains a reasonable curative approach for small and technically resectable tumors, although it is associated with a 25% local recurrence rate, which can be successfully treated with subsequent radiation. Beyond these modalities, intralesional agents—such as corticosteroids or rituximab—and topical therapies including nitrogen mustard, imiquimod, or bexarotene may be successfully used [20,21,22]. Intralesional rituximab, typically administered in weekly doses of 5–30 mg, induces similar response rates to systemic administration while using significantly lower doses [23]. For patients with T3 tumors (generalized skin involvement), management options include radiotherapy across multiple fields or a “watch and wait” strategy for asymptomatic cases, as radiation has not been found inferior to multiagent chemotherapy [20]. However, the watch and wait approach necessitates close follow-up due to the dreaded risk of transformation to diffuse large B-cell lymphoma. Predominantly, single-agent rituximab or combination chemoimmunotherapy in extensive/refractory cases is indicated for patients with generalized skin-only disease (T3) who have symptomatic or refractory presentations, as well as those with confirmed extracutaneous (nodal or visceral) involvement [19]. Finally, patients with more extensive or generalized cutaneous involvement are effectively managed with systemic single-agent rituximab, while intensive multiagent chemotherapy (such as R-CHOP) is generally reserved for rare cases of systemic involvement and/or transformation [9].

## 3. T-Cell Cutaneous Lymphomas (TCCLs) and Lymphoproliferative Disorders

### 3.1. Current Classification of TCCL and T-LPD

The 5th edition of the WHO classification and the International Consensus Classification (ICC) represent a significant evolution in the diagnostic framework of primary cutaneous T-cell lymphoproliferative disorders. A pivotal change in this update is the abandonment of the collective term “primary cutaneous peripheral T-cell lymphoma, rare subtypes,” in favor of listing these conditions as separate, specific entities. Consequently, several previously provisional categories—including primary cutaneous gamma/delta T-cell lymphoma, primary cutaneous CD8-positive aggressive epidermotropic cytotoxic T-cell lymphoma, and primary cutaneous CD4-positive small or medium T-cell lymphoproliferative disorder—have been upgraded to definite diagnostic entities. Central to this reclassification is the strategic transition from “lymphoma” to “lymphoproliferative disorder” (LPD) for certain indolent conditions, most notably primary cutaneous acral CD8-positive T-cell LPD. This terminological shift, supported by extensive clinical data, highlights the characteristically benign course, excellent prognosis and low risk of dissemination of the disease and serves as a vital clinical guide to prevent unnecessary aggressive systemic interventions. However, despite these refined diagnostic criteria, a profound disparity remains in our understanding of the molecular foundations of these rare entities [Table 4].

#### 3.1.1. Mycosis Fungoides

The current classification of cutaneous T-cell lymphomas maintains Mycosis Fungoides (MF) and its well-defined subtypes—folliculotropic MF (FMF), pagetoid reticulosis (PR), and granulomatous slack skin (GSS)—as central diagnostic entities. These subtypes significantly differ from classic disease. A clinical priority in FMF management is the rigorous differentiation between early-stage disease, which typically presents with indolent acneiform or comedo-like lesions, and advanced-stage disease characterized by dense cellular infiltrates and an impaired prognosis comparable to tumor-stage classic MF [8,24]. While PR remains a localized variant with an excellent prognosis, other forms such as granulomatous MF (GMF) exhibit sarcoidal infiltrative patterns and an adverse prognostic outlook, despite remaining classified under the broader MF umbrella due to histological overlaps with GSS [25,26]. Morphological pattern remains the main prognostic factor alongside the stage in clinic practice [25]. Beyond morphology, the genomic landscape of MF is increasingly defined by progression-associated alterations, including recurrent mutations in JAK3 and FGFR4, alongside novel gene rearrangements in ARID1A and CDKN2A/B. The identification of high-risk DNA methylation profiles in genes such as SOCS1 and NEUROG1, as well as the high mutational burden and PDL1 structural variants associated with large-cell transformation (LCT), is critical for early risk stratification. Early molecular detection of LCT is particularly paramount, as it represents a significant adverse prognostic turning point that necessitates the optimization of risk-stratified therapeutic interventions [27,28]. For diagnosis, now it is preferable to demonstrate clonal T-cell receptor rearrangements in skin biopsies, as they act as a strong supporting diagnostic factor. Although the variants of MF are well characterized, they are not recognized as distinct entities in either the WHO or ICC classifications [10]. International classifications retain these presentations as variants within the unified MF diagnostic spectrum, prioritizing risk-stratified and stage-based management, such as distinguishing early-stage from advanced-stage patterns in FMF over subtypes [10].

#### 3.1.2. CD30^+^ Cutaneous LPD

In the new structure of the WHO 5th edition, primary cutaneous anaplastic large-cell lymphoma (pcALCL) and lymphomatoid papulosis (LyP) are now distinct entities rather than subtypes [4]. pcALCL has an unique tumor site predilection and has a much more favorable prognosis than its systemic counterpart; therefore, this led to separate classifications for these two entities [2]. Although *ALK*-positive primary cutaneous lymphomas share similar pathological features with pcALCL, they are not grouped separately [29]. Primary cutaneous *ALK*-positive anaplastic large-cell lymphoma (pcALCL) carries an excellent prognosis, especially in pediatric populations, with surgical excision and/or radiotherapy serving as the standard therapeutic modalities. Nevertheless, rigorous long-term surveillance is warranted, as adult patients remain at risk for cutaneous relapse and systemic progression [30].

#### 3.1.3. Primary Cutaneous Peripheral T-Cell Lymphoma, Not Otherwise Specified (pcPTCL, NOS)

pcPTCL, NOS typically has an aggressive clinical course characterized by rapidly progressive tumors that frequently ulcerate and a poor five-year survival rate of approximately 54% [31]. Clinically, it typically presents with fast growing, solitary or disseminated tumors, often localized to the extremities. Histologically, it demonstrates a predominantly diffuse or nodular infiltrate, frequently extending into the subcutaneous tissue, with absent or only mild and focal epidermotropism. Immunophenotypically, pcPTCL-NOS frequently exhibits non-canonical T-cell profiles—such as double-negative (CD3+CD4−CD8−) or double-positive (CD3+CD4+CD8+) phenotypes—along with frequent aberrant expression of B-cell markers such as CD20 (observed in ~18% of cases) and strong *GATA-3* positivity in limb-localized solitary tumors. pcPTCL-NOS has been officially introduced as a distinct entity in a separate chapter of the 5th edition of the WHO classification. This classification integrates clinical, histopathological, immunophenotypic, and molecular findings rather prioritizing clinical behavior. The ICC has not recognized pcPTCL-NOS as a distinct entity yet [4,5,31].

#### 3.1.4. Other PTCL/LPD

The fundamental rationale for the latest classification updates in the 5th edition of the WHO and the International Consensus Classification (ICC) is the understanding of the importance of clinical behavior over histomorphological appearance in indolent conditions. Entities such as primary cutaneous CD4-positive small/medium T-cell lymphoproliferative disorder (pcSMTLPD) and primary cutaneous acral CD8-positive T-cell LPD have transitioned from the “lymphoma” to “lymphoproliferative disorder”. This reclassification is particularly critical for pcSMTLPD; by restricting the diagnosis to solitary nodules on the head, neck, or upper trunk, clinicians can maintain a 100% five-year survival rate, thereby protecting patients from invasive staging investigations and unnecessary and toxic systemic chemotherapy [19]. Furthermore, these updates recognize the importance of distinguishing these indolent LPDs from their aggressive counterparts and secondary cutaneous involvements through refined immunophenotypic and genetic markers. Indolent entities, such as primary cutaneous CD4-positive small/medium T-cell lymphoproliferative disorder (PCSM-TLPD), characteristically exhibit a T-follicular helper immunophenotypic profile (including expression of markers such as PD-1), whereas primary cutaneous acral CD8-positive lymphoproliferative disorder presents with a localized, non-destructive CD8-positive phenotype. In contrast, aggressive cutaneous lymphomas are defined by distinct, cytotoxic, and invasive programs; primary cutaneous gamma/delta T-cell lymphoma (PCGD-TCL) is identified by mature TCR γ/δ chain expression, while primary cutaneous CD8-positive aggressive epidermotropic cytotoxic T-cell lymphoma is distinguished by prominent epidermotropism and an aggressive cytotoxic phenotype [4,5,32,33]. Primary cutaneous acral CD8+ LPD exhibits an excellent prognosis despite having histological features of a lymphoma, requiring differentiation from aggressive CD8+ variants through its low proliferative activity and characteristic immunophenotype (dot-type CD68 positivity may assist in differentiating the diagnoses, but is not specific) [33]. Conversely, aggressive entities like primary cutaneous CD8+ aggressive epidermotropic cytotoxic T-cell lymphoma (AECTCL) are categorized as definite lymphomas due to their rapid evolution and specific genetic drivers (*JAK2*, *NFkB* and *CDKN2A/B* alterations). These molecular hallmarks not only serve as diagnostic markers but also broaden the therapeutic spectrum by identifying candidates for targeted interventions [4,10].

### 3.2. Current Management of CTCL and T-LPD

#### 3.2.1. Mycosis Fungoides

Currently, the management of MF is largely driven by disease stage. The main goal of the treatment is improving quality of life while avoiding treatment-related toxicity. For earlier-stage disease, local treatments are frequently employed, unlike advanced disease which is often treated with systemic therapies.

Local treatments include topical corticosteroids and phototherapy options such as narrowband UVB or psoralen plus ultraviolet A (PUVA). Topical steroids are particularly effective for patchy disease. Other localized options are scarcely utilized compared with steroids and phototherapy. Topical mechlorethamine (nitrogen mustard) and localized radiotherapy for recalcitrant lesions have substantial efficacy [34,35,36].

For patients with advanced-stage disease (IIB-IV) or those whose skin disease is refractory to topical treatments, systemic therapies are generally employed, often in an escalating fashion. Common systemic options include biologic response modifiers such as interferon-alpha and oral retinoids like bexarotene and/or low-dose methotrexate [37,38,39]. Recent therapeutic advances have introduced targeted antibody therapies, including brentuximab vedotin for CD30-expressing lesions and mogamulizumab, which targets the CCR4 receptor [40,41]. Histone deacetylase (HDAC) inhibitors like vorinostat and romidepsin also provide effective disease control like other hematological malignancies. These agents can be used to treat different compartments simultaneously, including the skin and blood (Table 5) [42].

#### 3.2.2. Early-Stage MF

Early-stage Mycosis Fungoides (MF), which includes stages IA, IB, and IIA, is characterized by a non-aggressive clinical course with a normal life expectancy for most patients. Stage IA is defined by patches or plaques covering less than 10% of the body surface area (BSA). Stage IB involves 10% or more BSA, whereas stage IIA involves skin involvement accompanied by enlarged but histologically uninvolved lymph nodes. The primary therapeutic goal for these patients is to attain disease control and ensure quality of life. Skin-directed therapies (SDTs) are viewed as the first-line standard of care. These localized treatments typically include potent topical corticosteroids, phototherapy (narrow-band UVB or PUVA), and topical chemotherapy such as mechlorethamine gel [8,43].

While some expert platforms have noted that watch and wait may be suitable for early-stage disease, the general consensus favors SDT [8,44]. Topical corticosteroids are the most frequently utilized first-line skin-directed therapy for patients with early-stage Mycosis Fungoides (IA–IIA) [44]. These agents exert their therapeutic effect through potent anti-inflammatory, immunosuppressive, and lymphocytotoxic mechanisms, which directly target the malignant T-cell clones in the dermis and epidermis [34]. Mechlorethamine 0.02% gel is an established topical alternative. Data from a phase 2 trial involving patients with stage IA-IIA MF indicated that daily application for up to one year resulted in overall and complete responses in 58.5% and 13.8% of patients [45]. It is highly recommended that high-potency topical steroids be used in conjunction with an expert dermatologist.

For steroid-refractory cases, bexarotene 1% gel is the only FDA-approved topical retinoid, achieving an ORR between 44% and 63% depending the disease’s refractoriness profile [46]. Clinical evidence suggests that treatment-naive patients may experience higher success rates compared to those previously treated with other topicals (75% vs. 67%), with a median response duration of nearly 2 years [46]. Other topical options include tazarotene 0.1% as an adjuvant and carmustine, which remains effective for patch/plaque MF, with response rates approaching 92% in T1 patients [47]. Toll-like receptor (TLR) agonists like imiquimod 5% and resiquimod have also shown activity; imiquimod achieved an 80% ORR and 45% CR in early-stage disease, while resiquimod have achieved clinical improvement in 75% of lesions with a reduction in malignant T-cell clones [48,49].

Phototherapy is a vital modality often used alone or in combination with topicals, with narrowband UVB (311 nm) favored for patch and plaque stages and PUVA remaining the modality of choice for patients with widespread skin involvement. Given the radiosensitivity of MF, radiation is utilized for curative intent in localized disease, while advanced skin disease may derive benefits from palliative local radiation or total skin electron beam therapy (TSEBT). Low-dose and conventional TSEBT (10–12/30–36 Gy) over conventional doses results in comparable ORRs of 87–98%, while significantly reducing short-term complications and facilitating future re-treatment. Low-dose TSEBT demonstrates a longer progression-free survival (PFS) than more advanced disease (27 vs. 11 months) [8,50,51,52].

#### 3.2.3. Advanced-Stage MF

The systemic management of advanced MF follows a risk-adapted approach that prioritizes immunomodulatory and biological response modifiers. Bexarotene, a selective retinoid X receptor agonist, is a primary systemic option with overall response rates (ORRs) of approximately 45% at a target dose of 300 mg/m^2^/day. Central hypothyroidism and hypertriglyceridemia are the most expected toxicities and patients typically treated prophylactically with levothyroxine and fenofibrate [53]. Interferon-alpha offers ORRs between 50% and 70%, and is frequently combined with phototherapy or extracorporeal photopheresis (ECP) to enhance efficacy. Additionally, low-dose methotrexate may be considered as an effective option for non-erytrodermic patients, yielding an ORR of roughly 40%, although it requires careful monitoring for hepatotoxicity and dose adjustments for renal impairment [7,8].

Extracorporeal photopheresis (ECP) is widely regarded as a first-line systemic modality for erythrodermic disease due to its ability to induce host anti-tumor immunity with minimal toxicity. ECP achieves response rates of approximately 60%, with complete responses in 20% of cases and a median response duration of two years [54]. ECP is most appropriate when used as a single therapy modality in low-tumor-burden SS together with erythrodermic MF [19]. Histone deacetylase (HDAC) inhibitors works by inducing epigenetic repression of gene transcription, differentiation and apoptosis induction. Romidepsin has demonstrated an ORR of 38% in advanced-stage disease, providing rapid symptom relief and durable remissions that can sometimes be maintained with attenuated dosing schedules [8].

The introduction of targeted biologics represents a significant leap in the treatment of refractory cases, as seen with the humanized anti-CCR4 antibody mogamulizumab and the anti-CD30 antibody–drug conjugate brentuximab vedotin. Mogamulizumab demonstrated a superior progression-free survival (PFS) of 7.7 months compared to 3.1 months for vorinostat in the phase III MAVORIC trial, showing particularly superior activity in the blood compartment, with a 68% ORR. A unique side effect is the mogamulizumab-associated rash (MAR), which can mimic CTCL and may complicate clinical/histological evaluation [54]. For patients with CD30 expression or large-cell transformation (LCT), brentuximab vedotin achieved an ORR of 60% and a median PFS of 16.1 months in the ALCANZA study [55]. Notably, brentuximab’s efficacy is not strictly dependent on high CD30 immunohistochemistry levels, making it broadly available for advanced MF [55]. The NCCN guidelines indicate that mogamulizumab is more preferable for erythrodermic Mycosis Fungoides and its efficacy does not require CD30 expression, whereas its role in large-cell transformation (LCT) is limited [19]. Conversely, brentuximab vedotin is a preferred systemic agent for CD30-positive Mycosis Fungoides, tumor-stage disease, and MF with large-cell transformation (MF-LCT). Thus, therapeutic selection between these two targeted agents is fundamentally dictated by the presence of tumor burden versus CD30 positivity and transformed large-cell phenotype [19]. Response to brentuximab vedotin is not clearly correlated with either CD30-positive cell percentage or CD30 density [19].

Emerging horizons in immunotherapy and curative strategies are reshaping long-term expectations for high-risk CTCL patients. Denileukin diftitox-cxdl (E7777), a refined IL-2 receptor-targeted fusion protein, was licensed in 2024 for stage I-III CTCL following phase III data indicating an ORR of 44% to 47.5% and a median PFS greater than two years [56]. Checkpoint blockade using agents like pembrolizumab has also shown promise, with an ORR of 38% in heavily pretreated patients, suggesting that harnessing host immunity is viable even in genetically complex cases [57]. For younger, fit patients with refractory advanced disease, allogeneic hematopoietic stem-cell transplantation (allo-HSCT) remains the only curative alternative. Prospective evidence from the CUTALLO trial confirms that allo-HSCT significantly prolongs overall survival (median 51.5 months vs. not reached) for high-risk patients who achieve remission prior to the transplant [58].

#### 3.2.4. CTCLs Other than MF

In the landscape of non-Mycosis Fungoides (non-MF) cutaneous T-cell lymphoproliferative disorders, recent management strategies prioritize the prevention of overtreatment while sustaining disease control [3]. The 5th edition of the World Health Organization (WHO) classification upgraded previous provisional entities to definite categories and set a conservative nomenclature, preferring the term lymphoproliferative disorder over lymphoma for entities with indolent behavior, such as primary cutaneous CD4-positive small or medium T-cell LPD and primary cutaneous acral CD8-positive LPD [4,5,10]. Given their highly favorable prognosis and limited risk of being advanced disease, skin-directed or minimally invasive strategies are still recommended as first-line options [32]. Specifically, for localized primary cutaneous CD4-positive small/medium T-cell LPD, modern de-escalated radiation therapy (RT) delivering an ultra-low dose of 4 Gy in two fractions has emerged as an exceptionally effective approach, eliciting complete response rates exceeding 92% with minimal toxicity and rare local relapses [59,60].

For patients presenting with more aggressive entities, localized multifocal lesions, or refractory disease, the therapeutic paradigm transitions to a response-adapted, multimodal algorithm incorporating targeted immunotherapy and systemically active agents. In primary cutaneous anaplastic large-cell lymphoma (pcALCL), local radiotherapy at a dose of 24 Gy in eight fractions serves as a highly effective standard for solitary lesions, whereas a lower palliative dose of 8 Gy in two fractions can rapidly alleviate symptoms in cases with multiple lesions [61]. In general, RT dose depends on curative versus palliative intent, lesion size, and disease distribution. When pcALCL progresses to systemic or visceral involvement, or in heavily pretreated refractory cases, targeted therapies like the antibody–drug conjugate brentuximab vedotin—which targets CD30-positive malignant cells—provide substantial systemic efficacy, demonstrating a superior objective response rates and significantly prolonged progression-free survival compared to conventional physician’s choice [55]. Furthermore, the investigation of immune checkpoint inhibitors targeting the PD-1/PD-L1 pathway, such as atezolizumab, continues to expand within the tumor microenvironment (TME) of refractory cutaneous T-cell malignancies. However, single-agent anti-PD-1/PD-L1 therapies have demonstrated relatively moderate or limited overall activity in pretreated advanced cohorts, and caution must be exercised due to the lack of reliable response predictors and the documented risk of eliciting hyperprogression or enhanced proliferation of malignant T-cells in specific biological subsets [57,62]. Although this phenomenon is mainly observed in Sezary syndrome, and the effectiveness of these agents has been taken into consideration, transient symptom worsening must be carefully differentiated from true malignant T-cell proliferation before prematurely discontinuing therapy [57,62].

## 4. Conclusions

Primary cutaneous lymphomas and lymphoproliferative disorders encompass a heterogeneous spectrum of diseases requiring tailored, risk-adapted management. Recent revisions in the WHO 5th edition and ICC 2022 classifications prioritize clinical behavior by reclassifying indolent entities—such as acral CD8+ and CD4+ small/medium T-cell proliferations—as lymphoproliferative disorders rather than overt lymphomas, thereby playing a pivotal role in preventing overtreatment and unnecessary staging. Parallel to these diagnostic revisions, therapeutic paradigms have bifurcated. Indolent subtypes and early-stage Mycosis Fungoides are predominantly managed with toxicity-sparing approaches, such as skin-directed therapies and ultra-low-dose radiotherapy (4 Gy), which achieve excellent disease control. In contrast, aggressive entities and advanced disease increasingly benefit from precision medicine, incorporating targeted biologics, antibody–drug conjugates (e.g., brentuximab vedotin, mogamulizumab), and immunotherapies. Aligning contemporary molecular diagnostic frameworks with personalized, risk-stratified therapies is still crucial for improving survival while optimizing patient quality of life.

## Figures and Tables

**Table 1:** Summary of the contemporary classification of CBCL.

Disease	Recent Revision
PCFCL	WHO 5th: Moved from follicular lymphoma subgroup to unique primary cutaneous follicular cell lymphoma subgroup; name is unchanged ICC 2022: Still under “follicular lymphomas” section
PCMZL	WHO 5th: Moved from “extranodal marginal zone lymphoma of mucosa-associated lymphoid tissue” section to marginal zone lymphoma; name is unchanged ICC 2022: Now describes the PCMZL as a cutaneous lymphoproliferative disorder rather than a lymphoma
PCDLBCL-LT	WHO 5th: Unchanged ICC 2022: Unchanged
EBV-positive mucocutaneous ulcer	WHO 5th: Unchanged ICC 2022: Unchanged
Inborn error of immunity-associated lymphoid proliferations and lymphomas	WHO 5th: Under “Lymphoid proliferations and lymphomas associated with immune deficiency and dysregulation” section; previously “Lymphoproliferative diseases associated with primary immune disorders” ICC 2022: Not recognized

**Table 2:** TNM classification of cutaneous lymphomas other than Mycosis Fungoides/Sezary syndrome.

T (tumor diameter)
T1: Solitary skin involvement T1a: solitary lesion < 5 cm diameter; T1b: solitary lesion ≥ 5 cm diameter
T2: regional skin involvement: multiple lesions limited to one body region or two contiguous body regions T2a: all disease encompassing a <15 cm diameter circular area T2b: all disease encompassing a 15 to <30 cm diameter circular area T2c: all disease encompassing a ≥30 cm diameter circular area
T3: generalized skin involvement T3a: multiple lesions involving two non-contiguous body regions T3b: multiple lesions involving ≥3 body regions
N (nodal involvement)
N0: No clinical or pathologic lymph node involvement N1: Involvement of one peripheral lymph node region (Ann Arbor system) that drains an area of current or prior skin involvement; biopsy positive for lymphoma N2: Involvement of two or more peripheral lymph node regions or involvement of any lymph node region that does not drain an area of current or prior skin involvement; biopsy positive for lymphoma N3: Involvement of central lymph nodes; biopsy positive for lymphoma Nx: Clinically abnormal peripheral or central lymph node but no pathologic determination
M (visceral involvement)
M0: No visceral involvement M1: Visceral involvement Mx: Visceral involvement is neither confirmed nor refuted by available pathologic or imaging assessment

**Table 3:** General treatment approaches for BCCL based on tumor characteristics.

Clinical Stage/Presentation	Recommended Approaches
Solitary Lesions	Low-dose radiotherapy Local radiotherapy Surgical excision Intralesional and topical therapy
T3/Asymptomatic Tumors	Multi-field radiotherapy Watch and wait
Extensive/Generalized Skin Involvement	Systemic therapy with single-agent rituximab

**Table 4:** Cutaneous T-cell lymphomas and lymphoproliferative diseases from EHO-EORTC 2018 Classification to WHO5th-ICC 2022.

WHO-EORTC 2018 4th Edition	Recent Revision
Mycosis Fungoides	WHO 5th: Unchanged ICC 2022: Unchanged
Mycosis Fungoides Variants Folliculotropic MF Pagetoid reticulocytosis Granulomatous slack skin	WHO 5th: “subtype” is used instead “variants”, ICC 2022 unchanged Granulomatous slack skin is not recognized in ICC 2022
CD30 LPD pcALCL LyP	WHO 5th: Each entity recognized as distinct entities; the umbrella title “CD30 positive lymphoproliferative diseases” has been removed ICC 2022: Unchanged
SPTCL	WHO 5th: Unchanged ICC 2022: Unchanged
Pc gamma-delta T-cell lymphoma	WHO 5th: Unchanged ICC 2022: Unchanged
CD8 AETCL Pc CD4+ SPT LPD Pc acral CD8+ T-cell lymphoma (all provisional)	WHO 5th: CD8 + AETCL (unchanged), pcCD4+ SMT LPD, pc acral CD8+ T-cell LPD ICC 2022: Same as WHO 5th
None	WHO 5th: pcPTCL-NOS ICC 2022: None

**Table 5:** General approach to MF.

Stage/Clinical Status	Preferred Approach	Key Agents/Modalities
**Limited (IA-IIA)**	Skin-Directed (Local)	Corticosteroids, Mechlorethamine, UVB/PUVA, Radiotherapy
**Advanced (IIB-IV)**	Systemic (Whole Body)	Retinoids, Interferon, Methotrexate
**Targeted/Refractory**	Biologic/Antibody	Mogamulizumab, Brentuximab, HDAC Inhibitors

## Data Availability

No new data were created or analyzed in this study. Data sharing is not applicable to this article.
